# Intravenous non-high-dose pantoprazole is equally effective as high-dose pantoprazole in preventing rebleeding among low risk patients with a bleeding peptic ulcer after initial endoscopic hemostasis

**DOI:** 10.1186/1471-230X-12-28

**Published:** 2012-03-28

**Authors:** Chih-Ming Liang, Jyong-Hong Lee, Yuan-Hung Kuo, Keng-Liang Wu, Yi-Chun Chiu, Yeh-Pin Chou, Ming-Luen Hu, Wei-Chen Tai, King-Wah Chiu, Tsung-Hui Hu, Seng-Kee Chuah

**Affiliations:** 1 Division of Hepatogastroenterology, Department of Internal Medicine, Kaohsiung Chang Gang Memorial Hospital, 123 Ta-Pei Road, Niaosung Hsiang, Kaohsiung City 833, Taiwan; 2 Division of Hepatogastroenterology, Department of Internal Medicine, Kaohsiung Chang Gung Memorial Hospital, Chang Gung University College of Medicine, Kaohsiung, Taiwan

**Keywords:** Intravenous proton-pump inhibitors, Peptic ulcer bleeding, Endoscopic hemostasis, Rebleeding, Rockall scores

## Abstract

**Background:**

Many studies have shown that high-dose proton-pumps inhibitors (PPI) do not further reduce the rate of rebleeding compared to non-high-dose PPIs but we do not know whether intravenous non-high-dose PPIs reduce rebleeding rates among patients at low risk (Rockall score < 6) or among those at high risk, both compared to high-dose PPIs. This retrospective case-controlled study aimed to identify the subgroups of these patients that might benefit from treatment with non-high-dose PPIs.

**Methods:**

Subjects who received high dose and non-high-dose pantoprazole for confirmed acute PU bleeding at a tertiary referral hospital were enrolled (n = 413). They were divided into sustained hemostasis (n = 324) and rebleeding groups (n = 89). The greedy method was applied to allow treatment-control random matching (1:1). Patients were randomly selected from the non-high-dose and high-dose PPI groups who had a high risk peptic ulcer bleeding (n = 104 in each group), and these were then subdivided to two subgroups (Rockall score ≥ 6 *vs*. < 6, n = 77 *vs*. 27).

**Results:**

An initial low hemoglobin level, serum creatinine level, and Rockall score were independent factors associated with rebleeding. After case-control matching, the significant variables between the non-high-dose and high-dose PPI groups for a Rockall score ≥ 6 were the rebleeding rate, and the amount of blood transfused. Case-controlled matching for the subgroup with a Rockall score < 6 showed that the rebleeding rate was similar for both groups (11.1% in each group).

**Conclusion:**

Intravenous non-high-dose pantoprazole is equally effective as high-dose pantoprazole when treating low risk patients with a Rockall sore were < 6 who have bleeding ulcers and high-risk stigmata after endoscopic hemostasis.

## Background

Acute, non-variceal upper gastrointestinal bleeding is a common cause of hospitalization and mortality has remained at 6% to 8% despite recent advances in both pharmacological and endoscopic therapy [1,2]. The risk of recurrent bleeding is increased in patients with high-risk stigmata found by endoscopy. Endoscopic hemostasis is able to control bleeding and reduce the rebleeding rate, morbidity and even mortality of this disease [3,4]. The success of hemostasis, however, is highly dependent on the intragastric pH and studies have shown that, when the intra-gastric pH is low, platelet function is impaired and pepsin is activated, which disaggregates platelet plugs [5,6]. The maintenance of an intragastric pH above 6.0 allows stabilization of the clot, which stops peptic ulcer (PU) bleeding and prevents rebleeding [7,8]. Interestingly, in Hung's study [9], the time during which a intragastric pH above 6 was maintained was similar for both a non-high dose PPI group and a high dose group (49%, 59%, *p *= 0.182). This poses the question as to what is the optimal dose of PPI that is able to achieve the required therapeutic goal, This continues to be a controversial issue in clinical practice. Both the Vienna and Asia-Pacific consensus recommend intravenous high-dose PPI therapy after successful endoscopic hemostasis; however, the evidence related to the use of low-dose PPIs is limited [10,11]. Many studies have shown that high-dose PPIs do not further reduce the rate of rebleeding compared to non-high-dose PPIs [12-14]. Nevertheless, we do not know whether the effect of intravenous non-high-dose PPIs is able to reduce the rebleeding rate among patients at low risk (Rockall score ≤ 6) or among those at high risk, both compared to high-dose PPIs. We can assume that intravenous high-dose PPI therapy is no doubt beneficial after successful endoscopic hemostasis but non-high dose treatment may be equally effective among certain subgroup of patients. If this is true, a change in strategy with respect to the use of PPIs may be both beneficial and cost-effective. The aim of this retrospective case-controlled study was to identify the subgroup of patients that might benefit from non-high-dose PPI treatment.

## Methods

### Study design

We reviewed 477 consecutive medical records of subjects with confirmed gastric and duodenal ulcers bleeding by endoscopic study between Jan. 2009 and March. 2011. All subjects received endoscopic hemostatic therapy for high-risk stigmata (active bleeding or a visible vessel in an ulcer bed) and were prescribed intravenous pantoprazole. The non-high-dose PPI patients were those who received an 80 mg pantoprazole bolus, which was followed by intravenous pantoprazole 80 mg per day until alimentation was possible, and then they received 40 mg per day pantoprazole orally. On the other hand, high-dose PPI patients received an 80 mg pantoprazole intravenous bolus injection, then were treated with 8 mg per hour continuous infusion of pantoprazole for 3 days, which was followed by intravenous 80 mg per day. A video endoscope (Olympus GIF-XQ240 or GIF-XQ 260, Tokyo, Japan) was used to perform the endoscopic therapy on all patients at our hospital. Individuals were excluded if the ulcer was malignant; there was non ulcerative bleeding such as angiodysplasia or a Mallory-Weiss tear, if the subject was lost to follow up before 30 days except due to mortality, and if the patient did not successfully undergo the initial endoscopic hemostasis. A total of 413 patients were enrolled for further analysis. For comparison, the patients were classified into two groups: subjects who achieved sustained hemostasis (n = 324) and those who did rebleed (n = 89). The patients' demographic and clinical characteristics were recorded including age, gender, initial shock status, red blood cell count, the amount of blood transfusion as packed red blood cells (PRBC), time to endoscope, underlying morbidities, drug use, such as non-steroid anti-inflammatory drugs (NSAIDs), aspirin, clopidogrel or warfarin and the Rockall score calculated as described in a previous study [15]. The characteristics of the ulcer, such as size, the presence of multiple ulcers in association, and the Forrest classification, which was determined as described in a previous study, were also investigated [16,17].

Next nearest neighbor matching was implemented using the greedy matching algorithm (NCSS 2007, Kaysville, Utah 84037, USA) to reduce bias in this retrospective study. This matching algorithm was performed to find equivalent matched controls in high-dose pantoprazole group for each individual in the non-high-dose group. The matching variables were stage of chronic kidney disease (CKD), Forrest classification and Rockall score. Effectively, patients were randomly selected to form non-high-dose and high-dose PPI groups that had a high risk PU bleeding (n = 104 in each group); these groups were then subdivided to two subgroups, namely those with a Rockall score of ≥ 6, n = 77 and those with a Rockall score of ≤ 6, n = 27). Risk stratification was defined according to the Rockall scoring system. Patients with a score ≧ 6 were considered high risk patients. Otherwise, they were classified as low risk patients as validated by Church and colleagues [18]. This study was approved in accordance with the principles of the Helsinki Declaration by both the Institutional Review Board and the Ethics Committee of Chang Gung Memorial Hospital, Taiwan (IRB 100-2003B).

### Definitions

The classification of chronic kidney disease was defined according to Kidney Disease: Improving Global Outcomes (KDIGO) [19]. The primary end points were rebleeding, need for surgery, and mortality. Rebleeding was defined based on the clinical physician's evaluation as ongoing melena passage, hematemesis, fresh blood or coffee-ground material in nasogastric tube or a decline in the hemoglobin level with or without PRBC transfusion. Shock was defined as tachycardia, a heart rate > 100/min, or hypotension with a systemic blood pressure < 90 mmHg [14,20].

High risk ulcers were defined according to the Forrest classification [16]. With respect to high-risk stigmata, active bleeding was defined as a continuous blood spurting (Forrest IA) or oozing (Forrest IB) from the ulcer base. A non-bleeding visible vessel at endoscopy was defined as a discrete protuberance at the ulcer base (Forrest IIA). An adherent clot was one that was resistant to forceful irrigation or suction (Forrest IIB). With respect to low-risk stigmata, a flat base, a pigmented spot or a clean base were defined as Forrest grade IIC or III.

### Statistical analysis

The Statistical Package for Social Sciences (SPSS15.0, Chicago, IL, USA) for Windows was used to analyze the data. The results are expressed as distributions, absolute frequencies, relative frequencies, medians and ranges, or means ± standard deviation (SD). The quantitative data were compared using the Student's *t*-test when the variables had a normal distribution. Differences between the proportions of categorical data were evaluated by Fisher's exact test when the number of expected subjects was less than five and otherwise by the χ^2 ^test. A multivariate logistic regression model was used to assess the independent association between the rebreeding and non-rebleeding groups. A *p *value of < 0.05 was considered statistically significant. Further statistical analysis was performed after case-controlled matching the subgroups with Rockall scores ≥ 6 or < 6 by the greedy methods in order to compare rebleeding between the high-dose and non-high-dose PPI groups.

## Results

Among the 477 subjects who had their health records reviewed, four subjects were lost to follow up before 30 days other than by death, two subjects failed their initial endoscopic hemostasis, thirteen suffered from angiodysplasia, twenty five had gastric cancers, seventeen had gastric antral vascular ectasia and three had a Mallory-Weiss tear; these individuals were all excluded and eventually 413 subjects were enrolled for further analysis. Among these 413 enrolled patients, 219 were treated by endoscopic epinephrine injection, clips or thermocoagulation alone and 194 were treated by clips, or thermocoagulation in combination with epinephrine injection. There were no significant differences between the two study groups with respect to sustained hemostasis *vs*. rebleeding groups in terms of age, drug use (such as NSAIDs, aspirin or warfarin), shock at presentation, time to endoscopy, ulcer size and stage of Forrest classification(Table 1). Significant differences were observed for variables such as gender (female: 26.8% *vs*. 39.3%, *p *= 0.022), creatinine (2.0 ± 2.3 g/L *vs*. 3.6 ± 3.3 g/L, *p *= 0.010), initial hemoglobin level (96.7 ± 28.2 g/L *vs*. 82.5 ± 24.1 g/L, *p *≤ 0.001), platelet count (197.4 ± 87.3 × 10^9^/L *vs*. 188.0 ± 129.0 × 10^9^/L, *p *= 0.031), CKD stage III to V (38.9% *vs*. 70.8%, *p *≤ 0.001), DM (26.9% *vs*. 41.6%, *p *= 0.007), Rockall score (6.0 ± 1.6 vs. 6.8, ± 17, *p *< 0.001), amount of blood transfused as PRBC (1203.2 ± 1640.4 mL *vs*. 2192.6 ± 2077.3 mL, *p *< 0.001), surgery (0.3 vs.7.9, *p *< 0.001), hospital stay (11.5 ± 13.5 vs. 29.9 ± 46.0, *p *< 0.001)and mortality (6.8% *vs*. 29.2%, *p *< 0.001). On multivariate analysis, an initial low hemoglobin level, serum creatinine level, and Rockall score were independent factors associated with rebleeding (Table 2).

**Table 1:** Basic demographic and clinical characteristics of all enrolled patients

Variables	Sustained Hemostasis (n = 324)	Rebleeding (n = 89)	*p*-value
**Age (years)**	64.8 ± 13.9	66.4 ± 13.2	0.812

**Female gender, n (%)**	87(26.8)	35(39.3)	0.022*

**Hb **(g/L)	96.7 ± 28.2	82.5 ± 24.1	< 0.001*

**Creatinine**	2.0 ± 2.3	3.6 ± 3.3	< 0.001*

**Platelet **(× 10^9^/L)	197.4 ± 87.3	188.0 ± 129.0	0.031*

**Use of NSAIDs, n (%)**	29(9.0)	4(4.5)	0.170

**Use of aspirin, n (%)**	60(18.5)	9(10.1)	0.060

**Use of clopidogrel, n (%)**	38(11.7)	11(12.4)	0.870

**Use of wafarin, n (%)**	15(4.6)	5(5.6)	0.700

**Shock on admission, n (%)**	165(50.9)	53(59.6)	0.149

**Coexisting illness, n (%)**			
**CKD III to V**	126(38.9)	63(70.8)	< 0.001*
**COPD**	22(6.8)	7(7.9)	0.725
**CAD**	62(19.1)	23(25.8)	0.166
**DM**	87(26.9)	37(41.6)	0.007*
**CVA**	54(16.7)	20(22.5)	0.206
**Liver Cirrhosis**	55(17.0)	12(13.5)	0.429

**Rockall score**	6.0 ± 1.6	6.8 ± 1.7	< 0.001*

**Time to endoscope (h)**	14.7 ± 13.6	18.5 ± 20.3	0.333

**Ulcer size (cm)**	1.0 ± 0.6	1.0 ± 0.6	0.884

**Multiple ulcers, n (%)**	105(32.4)	36(40.4)	0.156

**High stigmata in Forrest classification, n (%)**	316(97.5)	86(96.6)	0.640

**PRBC BT(mL)**	1203.2 ± 1640.4	2192.6 ± 2077.3	< 0.001*

**Surgery, n (%)**	1(0.3)	7(7.9)	< 0.001*

**Hospital stay(days)**	11.5 ± 13.5	29.9 ± 46.0	< 0.001*

**Mortality, n (%)**	22(6.8)	26(29.2)	< 0.001*
**Bleeding related/Other causes**	5/17	14/12	

**Abbreviations: ***OR*, odds ratio; *CI*, confidence interval; *Hb*, hemoglobin; *CKD*, chronic kidney disease; *NSAID*, nonsteriodal anti-inflammatory drug; *PPI*, proton-pump inhibitor; *DM*, diabetes mellitus type 2*; COPD*, chronic obstructive pulmonary disease; *CAD*, coronary artery disease; *CVA*, cerebrovascular accident; *BT*, blood transfusion. * *p *< 0.05

**Table 2:** Predictors of recurrent bleeding from stepwise logistic regression in the multivariate analysis

	Odds ratio	**95% CI**.	*p*-value
**Hb **(g/L)	0.877	0.791-0.972	0.012

**Creatinine**	1.144	1.050-1.247	0.002

**Rockall score**	1.201	1.021-1.412	0.027

**Abbreviation: ***Hb*, hemoglobin; **CI: Confidence interval**

The greedy method (NCSS 2007) was used to create treatment-control random matching of individuals based on stage of CKD and Rockall score, and 104 patients were randomly selected to form the non-high-dose and high-dose patient groups (Table 3). Among high risk patients (n = 77), the significant variables were the rebleeding rate (14.3% *vs*. 40.2%, *p *= 0.001), and amount of blood transfused as PRBC (1373.4 ± 1309.5 mL *vs*. 2539.0 ± 2271.1 mL, *p *≤ 0.001). On the other hand, among the low risk patients (Rockall score ≤ 6), the baseline demographic and clinical characteristics such as rebleeding rate (3/27, 11.1% in each group), surgical interventions, and mortality were similar for both the high-dose and non-high-dose groups (Table 4).

**Table 3:** Comparison between the non-high-dose and high-dose groups of patients after randomly matched analysis by using Greedy method

Characteristic	Non-high-dose Group (n = 104)	High-dose Group (n = 104)	*P*-value
**Age (years)**	65.6 ± 11.8	65.5 ± 13.9	0.258

**Female gender, n (%)**	24(23.1)	39(37.5)	0.024*

**Creatinine**(mg/dl)	2.4 ± 2.3	2.8 ± 2.9	0.010*

**Hb **(g/L)	88.9 ± 25.2	89.1 ± 27.6	0.386

**Platelet**(× 10^9^/L)	191.5 ± 88.8	198.9 ± 106.3	0.113

**Use of NSAIDs, n (%)**	9(8.7)	11(10.6)	0.638

**Use of aspirin, n (%)**	15(14.4)	18(17.3)	0.569

**Use of clopidogrel, n (%)**	11(10.6)	13(12.5)	0.664

**Use of wafarin, n (%)**	6(5.8)	10(9.6)	0.298

**Shock at presentation**	52(50.0)	65(62.5)	0.069

**Coexisting illness, n (%)**			
**CKDIII, IV/V**	40/14 (38.5/13.5)	40/15(38.5/14.4)	0.978
**COPD**	7(6.7)	6 (5.8)	0.775
**CAD**	19(18.3)	28(26.9)	0.136
**DM**	34(32.7)	39(37.5)	0.468
**CVA**	21(20.2)	23(22.1)	0.734
**Liver Cirrhosis**	20(19.2)	14(13.5)	0.261

**Rockall score**	6.4 ± 1.4	6.4 ± 1.4	1.000

**Time to endoscope (h)**	16.4 ± 20.3	16.9 ± 19.8	0.482

**PRBC BT(mL)**	1252.4 ± 1272.1	2156.3 ± 2117.1	0.007

**Ulcer size (cm)**	1.0 ± 0.6	0.9 ± 0.6	0.901

**Multiple ulcers, n (%)**	29(27.9)	38(36.5)	0.182

**Forrest classification Ia/Ib/IIa/IIb/IIc/III**	2/70/6/26/0/0	3/70/6/25/0/0	0.974

**Re-bleeding, n (%)**	14 (13.5)	34 (32.7)	0.001*

**Surgery, n (%)**	2(1.9)	1(1.0)	0.561

**Hospital stay (days)**	13.9 ± 22.3	17.9 ± 15.3	0.641

**Mortality, n (%)**	10(9.6)	16(15.4)	0.403
**Bleeding related/Other causes**	4/6	8/8	

**Abbreviation: ***Hb*, hemoglobin; *NSAID*, nonsteriodal anti-inflammatory drug; *CKD*, chronic kidney disease; *PPI*, proton-pump inhibitor; *DM*, diabetes mellitus type 2*; COPD*, chronic obstructive pulmonary disease; *CAD*, coronary artery disease; *CVA*, cerebrovascular accident; *BT*, blood transfusion. * *p *< 0.05

**Table 4:** Comparison between the intravenous non-high-dose and high-dose PPI after case-controlled matching for the subgroup of low risk patients (Rockall score < 6)

Characteristics	Non-high-dose Group (n = 27)	High-dose Group (n = 27)	*p*-value
**Age (years)**	65.8 ± 3.8	62.4 ± 14.7	0.991

**Female gender, n (%)**	4(14.8)	10(37.0)	0.119

**Creatinine**(mg/dl)	1.3 ± 0.5	1.5 ± 1.5	0.100

**Hb **(g/L)	95.4 ± 25.1	106.0 ± 29.9	0.340

**Platelets **(× 10^9^/L)	202.2 ± 81.1	246.5 ± 91.6	0.151

**Use of NSAIDs, n (%)**	3(11.1)	5(18.5)	0.704

**Use of aspirin, n (%)**	2(7.4)	1(3.7)	1.000

**Use **of **clopidogrel, n (%)**	1(3.7)	2(7.4)	1.000

**Use of wafarin, n (%)**	1(3.7)	3(11.1)	0.610

**Shock at presentation**	9(33.3)	11(40.7)	0.573

**Coexisting illness, n (%)**			
**CKD III, IV and V**	7(25.9)/0	7(25.9)/0	1.000
**COPD**	1(3.7)	0	1.000
**CAD**	2(7.4)	2(7.4)	1.000
**DM**	7(25.9)	8(29.6)	0.761
**CVA**	3(11.1)	5(18.5)	0.704
**Liver Cirrhosis**	1(3.7)	1(3.7)	1.000

**Rockall score**	4.5 ± 0.6	4.5 ± 0.6	1.000

**Time to endoscopy (hours)**	10.4 ± 11.3	10.6 ± 9.9	0.904

**PRBC BT(mL)**	907.4 ± 1109.7	1064.8 ± 1003.8	0.863

**Ulcer size (cm)**	1.1 ± 0.7	0.9 ± 0.6	0.506

**Multiple ulcers, n (%)**	7(25.9)	9(33.3)	0.182

**Forrest classification Ia/Ib/IIa/IIb**	0/18/2/7	0/18/2/7	1.000

**Re-bleeding, n (%)**	3(11.1)	3 (11.1)	1.000

**Surgery, n (%)**	0	0	1.000

**Hospital stay (days)**	5.7 ± 5.6	11.7 ± 8.6	0.031*

**Mortality, n (%), bleeding related and other causes**	0/0	0/1 (3.7)	1.000

***Abbreviation: ***Hb, hemoglobin; NSAID, nonsteriodal anti-inflammatory drug; CKD, chronic kidney disease; PPI, proton-pump inhibitor; DM, diabetes mellitus type 2; COPD, chronic obstructive pulmonary disease; CAD, coronary artery disease; CVA, cerebrovascular accident; BT, blood transfusion. * p < 0.05

## Discussion

There have been a number of studies in the literature that have reported there to be no difference in the magnitude of risk reduction between intensive high-dose and non-high-dose intravenous PPI therapy and these include those conducted by the Hung and Yuksel groups [9,21]. In addition, meta-analysis by Wang and Wu came to a similar conclusion, namely that low-dose intravenous PPI can achieve the same efficacy as high-dose PPI following endoscopic hemostasis [22,23]. A more recent prospective study conducted by Songür and colleagues also showed that high-dose esomeprazole infusion therapy following endoscopic hemostasis treatment is not superior to low-dose PPI therapy in the terms of re-bleeding, need for surgery and mortality [24]. Moreover, Chen and colleagues from Taiwan concluded in a randomized clinical trial that PPI dosage is not associated with rebleeding following combined endoscopic haemostasis of bleeding ulcers [25]. Obviously, the findings remain somewhat unconvincing with respect to the optimal dosage of PPI for ulcer bleeding despite the publication of updated Vienna and Asian-pacific consensus statements [10,11]. In parallel with the above findings, we still do not know whether the effect of intravenous non-high-dose PPIs is as good as high-dose PPIs in terms of reducing rebleeding rates among patients at low risk (Rockall score ≤ 6) or among those at high risk (Rockall score ≥ 6).

The overall rebleeding rate in current study was high at 21.5% (89/413) with Hung and Yuksel reported rebleeding rates of 3.9% (4/103) and 7.2% (7/97), respectively [9,21]. The differences can be explained by the enrollment in the present study of more patients with concurrent illness resulting in a mean Rockall score of 6.1. The prevalence of CKD in Yuksel's study was only 2.06% (2/97), and the mean score of American Society of Anesthesiologist (ASA) criteria was only 1.5 to 1.6 in Hung's study. As is well known, severe concurrent illness may dilute the results attained when assessing the effect of high-dose PPI treatment. All these studies, including ours, observed that intravenous high-dose pantoprazole treatment did not appear to be more effective at reducing rebleeding compared to a non-high-dose regimen among patients with high-risk stigmata. The greatest limitation with a retrospective case-controlled study like the current study is that, despite the attempt to minimize the possible selection bias between the two treatment groups by conducting greedy matching, prejudice is still inevitable among the high-dose group. For instance, there were still more rebleeding patients in the high risk patients with Rockall score ≥ 6 (14.3% *vs*. 40.2%, *p *= 0.001).

Most interestingly, after case-controlled matching, the low risk patient subgroup (Rockall score ≤ 6) analysis actually showed that the rebleeding rate was similar for both the high-dose and non-high-dose groups (3/27, 11.1% in each group) (Table 4). This implies that non-high-dose intravenous PPI is enough to preventing rebleeding among low risk patient. Theoretically, there is no doubt about the effectiveness of the anti-secretory effect of high-dose PPI. Evidence points clearly towards the fact that continuous intravenous infusion of PPI is able to maintain an intragastric pH > 6 for 59% to 98% of the time during monitoring [26-28]. In contrast, other studies have shown that low-dose intravenous PPI can be as effective as a high-dose regimen at maintaining a consistent pH of around 4-6 [29,30]. Choi and colleagues reported that low-dose continuous infusion of pantoprazole (40 mg bolus followed by 4 mg/h) was able to maintain an intragastric pH > 6 in a manner similar to that of a high-dose group (80 mg bolus followed by 8 mg/h) among Korean patients with PU bleeding requiring endoscopic hemostasis [31]. At least two other meta-analysis studies have revealed that high-dose PPIs are not superior to non-high-dose PPIs in terms of reducing the rate of rebleeding, neither with respect to the need for surgical intervention, nor in terms of mortality after endoscopic treatment among patients with bleeding PUs. This suggests that the non-high dose PPI treatment strategy may provide an equal clinical benefit among ulcer bleeding patients [24,25,32]. Therefore, it makes sense that non-high-dose intravenous PPI might be sufficient to reduce the rebleeding of PUs after initial endoscopic hemostasis among Taiwanese, at least among the subgroup of low risk patients that have a Rockall score < 6.

Co-morbidities influence the rate of recurrent bleeding [33]. Recurrent PU bleeding may be prolonged in individuals with co-morbidities and therefore Cheng and colleagues suggested a extended low dose infusion of intravenous PPIs for up to 7-days may result in better control of recurrent bleeding of PUs [13]. For instance, the fact that patients have a more severe stage of kidney disease (CKD V) might be relevant to increased rebleeding [34]. This is consistent with the present study in that CKD stage III to V was found to be an influencing risk factor for recurrent bleeding on univariate analysis in spite of the fact that all subjects with end stage renal disease (ESRD) received heparin-free dialysis at our hospital. Uremic platelet function impairment may be responsible for this higher risk of ulcer rebleeding [35]. The reason behind uremic platelet dysfunction involving the interaction of von Willebrand factor (vWf) with various platelet membrane glycoproteins, namely Ib and IIb to IIIa, which do not normalize after dialysis [36].

From the cost-effectiveness point of view, Leontiadis and colleagues reported that high-dose PPI therapy is more expensive, and that non-high dose is relatively inexpensive [37]; however, Barkun and colleagues proved that high-dose intravenous esomeprazole strategy is more effective and less costly than a non-intravenous esomeprazole strategy [38].

Several limitations of this study must be recognized. Firstly, this retrospective analysis is dependent on the completeness of the medical charts. If the chart report of ulcer pattern was not complete, we reviewed the image obtained by endoscopy or watching the recorded video in order to record the ulcer characteristics. Therefore, the investigation reliability might vary in this area. Secondly, selection bias definitely existed among high-dose group due to the clinicians' decision with respect to the dosage used for the more severely ill patients, even though we attempted to minimize this selection bias using the greedy matching method after controlling the baseline conditions of the subjects. Thirdly, some patients were treated by endoscopic epinephrine injection alone, which was suboptimal. Therefore, the intention to compare the overall efficacies between the non-high-dose and high-dose PPIs is hampered by these limitations. However, the interesting part of this study remains our observed in terms of the low risk subgroup and treatment strategy.

## Conclusion

Intravenous non-high-dose pantoprazole is equally effective as high-dose pantoprazole when treating low risk patients with bleeding ulcers and high-risk stigmata after endoscopic hemostasis and who have a Rockall sore that is < 6.

## Abbreviations

PPI: Proton-pump inhibitor; PU: Peptic ulcer; CKD: Chronic kidney disease; PRBC: Packed red blood cell; NSAID: Non-steroid anti-inflammatory drugs; SD: Standard deviation; PM: Poor metabolizer; ESRD: End stage renal disease; vWf: von Willebrand factor; GP: Platelet membrane glycoproteins.

## Competing interests

The authors declare that they have no competing interests.

## Authors' contributions

CML and JHL participated in the design of the study, coordinated the study, performed the statistical analysis and wrote the manuscript. YHK, KLW, YCC, YPC, MLH, WCT, KWC, and THH participated in the design of the study and consulted on the statistical analysis. SKC consulted on design of the study and on the interpretation of results. All authors read and approved the final manuscript.

## Pre-publication history

The pre-publication history for this paper can be accessed here:

http://www.biomedcentral.com/1471-230X/12/28/prepub
